# Photocatalytic CO_2_ Reduction Using Zinc Indium Sulfide Aggregated Nanostructures Fabricated under Four Anionic Conditions

**DOI:** 10.3390/nano14141231

**Published:** 2024-07-20

**Authors:** I-Hua Tsai, Eric Wei-Guang Diau

**Affiliations:** 1 Department of Applied Chemistry, Institute of Molecular Science, National Yang Ming Chiao Tung University, 1001 Ta-Hseuh Rd., Hsinchu 300093, Taiwan; as3855841@gmail.com; 2 Center for Emergent Functional Matter Science, National Yang Ming Chiao Tung University, 1001 Ta-Hseuh Rd., Hsinchu 300093, Taiwan

**Keywords:** semiconductor photocatalysis, zinc indium sulfide, hydrothermal synthesis, Kubelka–Munk, photocatalytic CO_2_ reduction

## Abstract

Zinc indihuhium sulfide (ZIS), among various semiconductor materials, shows considerable potential due to its simplicity, low cost, and environmental compatibility. However, the influence of precursor anions on ZIS properties remains unclear. In this study, we synthesized ZIS via a hydrothermal method using four different anionic precursors (ZnCl_2_/InCl_3_, Zn(NO_3_)_2_/In(NO_3_)_3_, Zn(CH_3_CO_2_)_2_/In(CH_3_CO_2_)_3_, and Zn(CH_3_CO_2_)_2_/In_2_(SO_4_)_3_), resulting in distinct morphologies and crystal structures. Our findings reveal that ZIS produced from Zn(CH_3_CO_2_)_2_/In_2_(SO_4_)_3_ (ZIS-AceSO_4_) exhibited the highest photocatalytic CO_2_ reduction efficiency, achieving a CO production yield of 134 μmol g^−1^h^−1^. This enhanced performance is attributed to the formation of more zinc and indium vacancy defects, as confirmed by EDS analysis. Additionally, we determined the energy levels of the valence band maximum (VBM) and the conduction band minimum (CBM) via UPS and absorption spectra, providing insights into the band alignment essential for photocatalytic processes. These findings not only deepen our understanding of the anionic precursor’s impact on ZIS properties but also offer new avenues for optimizing photocatalytic CO_2_ reduction, marking a significant advancement over previous studies.

## 1. Introduction

Global warming is one of the significant issues that the entire world has had to carefully confront in recent years [1]. Effectively reducing greenhouse gas emissions and mitigating their subsequent impacts are challenges that scientists are devoted to addressing. Various methods for CO_2_ conversion have been explored, including thermal, electrochemical, and photocatalytic approaches. Thermal CO_2_ conversion typically involves high-temperature reactions, often with the aid of catalysts, to produce valuable chemicals and fuels. This method has shown considerable potential, especially in converting CO_2_ into syngas, methane, and methanol. For instance, recent studies have demonstrated efficient CO_2_ hydrogenation using advanced catalysts under thermal conditions, yielding high-value products like liquid fuels or long-chain hydrocarbon (C_5+_) [2]. However, the high energy requirements and potential environmental impact of the necessary heat are significant drawbacks. Electrochemical CO_2_ conversion, on the other hand, uses electrical energy to drive the reduction of CO_2_, often in aqueous solutions with the help of electrocatalysts. This method can operate under milder conditions compared to thermal conversion and allows for precise control over the reaction parameters [3]. Liquid plasma cracking technology is also one of the methods that use electricity as an energy source. Among them, decomposing ammonia water to produce hydrogen without producing CO_2_ is one of the current promising technologies [4]. Nonetheless, the efficiency of electrochemical conversion heavily depends on the electrode materials and the overall system design. Furthermore, these catalytic methods not only demand extra energy consumption but also stand in stark contrast to the principles of sustainable development and green chemistry.

Utilizing semiconductor materials for photocatalytic reactions is a clean, environmentally friendly solution. Photocatalytic reactions, as the name suggests, harvest solar energy as the driving force for reactions, requiring no additional energy to catalyze the reactants. This makes it a highly attractive approach for transforming greenhouse gases such as CO_2_ into high-value products like CO, formic acid, methanol, or methane or splitting water into H_2_ gas [5,6,7,8,9,10,11,12]. In recent years, numerous semiconductor materials with excellent photocatalytic properties have emerged, including titanium dioxide [13,14,15,16], metal/carbon quantum dots [17,18,19,20], and perovskite materials [21,22,23,24], making them applicable to address energy and environmental challenges. Among them, zinc indium sulfide (ZnIn_2_S_4_, abbreviated as ZIS) is one of the highly promising photocatalytic semiconductor materials [25,26,27,28,29,30] due to its simple synthesis, low cost, and minimal need for organic solvents in reactions and purification, along with excellent light absorption, good stability, and low toxicity, which make it a crucial research target for photocatalytic CO_2_ reduction.

Previous studies indicated that ZIS has been subjected to various improvement and optimization strategies, including heteroatom doping [31,32,33,34], atomic vacancies [35,36,37,38], and heterostructure formation [39,40,41,42]. However, discussions regarding the intrinsic properties of ZIS (such as structural properties, defect formation, bandgap, and band position) remain unclear. For example, in terms of structure, nanoscale ZIS samples tend to have larger surface areas, which are more favorable for photocatalytic reactions. Secondly, defect generation also contributes to the spatial separation of photogenerated charge carriers. Third, the bandgap and position also have a profound impact on photocatalytic efficiency. Changing the band position may give the photogenerated charge carriers a greater potential to react. Due to the simplicity of synthesis of ZIS, there is a wide range of precursor choices, where various anions can serve as precursors for ZIS. However, the profound effects of these anions on ZIS are not well understood. The impact of these diverse precursors on the structure, defects, bandgaps, and photocatalytic performance of ZIS samples needs to be thoroughly explored. Therefore, this study focuses on synthesizing ZIS via a hydrothermal method with four precursor species (trisodium citrate, thioacetamide, Zn^2+^, and In^3+^) and investigates the differences in photocatalytic performance under four different anionic environments by altering the composition of the ionic salts (ZnCl_2_/InCl_3_, Zn(NO_3_)_2_/In(NO_3_)_3_ Zn(CH_3_CO_2_)_2_/In(CH_3_CO_2_)_3_, and Zn(CH_3_CO_2_)_2_/In_2_(SO_4_)_3_); the corresponding photocatalysts are labelled ZIS-Cl, ZIS-NO_3_, ZIS-Ace, and ZIS-AceSO_4_, respectively. We found that introducing various anionic species can induce zinc and indium vacancy defects that help to improve the photocatalytic performance for CO_2_ reduction to produce CO and methane. Furthermore, the energy levels of the valance band maximum (VBM) and the conduction band minimum (CBM) were determined via UPS and absorption spectra via two different methods; the corresponding results are discussed to gain a deeper understanding of energy band alignment with respect to photocatalytic performance.

## 2. Materials and Methods

### 2.1. Chemicals

The chemicals used were zinc chloride (Sigma-Aldrich, St. Louis, MO, United State, reagent grade, ≥98%), zinc nitrate hexahydrate (Sigma-Aldrich, reagent grade, ≥98%), zinc acetate dihydrate (Sigma-Aldrich, reagent grade, ≥98%), indium chloride (Sigma-Aldrich, reagent grade, 98%), indium nitrate hydrate (Sigma-Aldrich, 99.9% trace metals basis), indium acetate (Thermo Scientific, Ward Hill, MA, United State, 99.99% metals basis), indium sulfate (Thermo Scientific, 99.99% metals basis), sodium citrate dihydrate (for analysis, ExpertQ^®^, ACS, ISO, Reag. Ph Eur, Sentmenat, Barcelona, Spain), thioacetamide (Sigma-Aldrich, reagent grade, 98%), ethanol (95%), and triethanolamine (puriss. p.a., St. Louis, MO, United State, ≥99% GC).

### 2.2. Synthesis of the Zinc Indium Sulfide (ZIS) Powders [43,44,45]

Synthesis of ZIS using chloride salts as precursors:

1. Preparation of Precursor Solution:
  (1) In a 250 mL beaker, 1.25 mmol of zinc chloride (ZnCl_2_), 2.5 mmol of indium chloride (InCl_3_), 3.4 mmol of sodium citrate (Na_3_C_6_H_5_O_7_), and 5 mmol of thioacetamide (TAA) were accurately weighed and added to 100 mL of deionized water.
  (2) The mixture was stirred vigorously using a magnetic stirrer for 30 min at room temperature to ensure complete dissolution of the precursors.
2. Hydrothermal Synthesis:
  (1) The precursor solution was transferred quantitatively to a 125 mL Teflon-lined stainless-steel autoclave to ensure efficient reaction conditions. The autoclave was sealed securely and placed in an oven preheated to 180 °C.
  (2) The reaction was allowed to proceed for 12 h under autogenous pressure to promote the nucleation and growth of ZIS nanoparticles. After the reaction time elapsed, the autoclave was allowed to cool naturally to room temperature overnight to avoid thermal shock.
3. Product Purification:
  (1) The resulting ZIS nanoparticles were harvested by centrifugation at 11,000× *g* for 20 min to separate them from the reaction mixture.
  (2) The supernatant was carefully decanted, and the ZIS precipitate was washed twice with a mixture of deionized water and anhydrous ethanol to remove any residual precursors or by-products.
  (3) After washing, the ZIS sample was dried in a vacuum oven at 60 °C for 12 h to remove any remaining solvent and ensure the stability of the nanoparticles.
4. Naming:
  (1) The final product obtained from this synthesis procedure will be designated as ZIS-Cl to denote its synthesis route using chloride salts as precursors.

Synthesis of ZIS using nitrate salts as precursors:

Replace ZnCl_2_ and InCl_3_ with zinc nitrate hexahydrate (Zn(NO_3_)_2_·6H_2_O) and indium nitrate hydrate (In(NO_3_)_3_·xH_2_O), respectively, while keeping the remaining steps unchanged. The final product will be named ZIS-NO_3_.

Synthesis of ZIS using acetate salts as precursors:

Replace ZnCl_2_ and InCl_3_ with zinc acetate dihydrate (Zn(CH_3_COO)_2_·2H_2_O) and indium acetate (In(CH_3_COO)_3_), respectively, while keeping the remaining steps unchanged. The final product will be named ZIS-Ace.

Synthesis of ZIS using acetate and sulfate salts as precursors:

Replace ZnCl_2_ and InCl_3_ with (Zn(CH_3_COO)_2_·2H_2_O) and indium sulfate (In_2_(SO_4_)_3_), respectively, while keeping the remaining steps unchanged. The final product will be named ZIS-AceSO_4_.

### 2.3. Characterizations

The morphology of the ZIS powder was characterized using a field emission scanning electron microscope (FESEM) offering flexible elemental analysis optimized for EDS (JEOL JSM-7401F SEM). The X-ray diffraction (XRD) patterns of the ZIS powder samples deposited on a quartz substrate were obtained using an X-ray diffractometer (Bruker AXS, D8 Advance) with Cu Kα radiation (λ = 154.18 pm). The chemical state, chemical shift, work function, and valence band maximum of the ZIS powder samples were identified using X-ray photoelectron spectroscopy (XPS) and ultraviolet photoelectron spectroscopy (UPS; Thermo Fisher Scientific ESCALAB Xi+). The absorption spectra were measured using a JASCO V-780 spectrophotometer equipped with an integrating sphere attachment. The absorbance of the ZIS samples was estimated using Equation (1). According to the absorption spectra, the Tauc plot [45] was used to estimate the bandgap of the ZIS samples through Equation (2). In addition, the other analytical method used to calculate the bandgap is the Kubelka–Munk model [46] using Equation (3). Upon using Equation (3), the ZIS bandgap can be estimated by another function via a K–M plot, which is a similar function to the Tauc plot. The K–M plot can be obtained via Equation (4), as shown below.

A = 1 − %R(1)

where A is the absorbance of the powder sample and %R is the reflectance of the powder sample collected from the UV-vis-NIR spectrophotometer.

(2) $${\left(\alpha h\nu \right)}^{2}=A\left(h\nu -E_{g}\right)$$

where α is the absorption coefficient of the ZIS samples, hν is the photon energy, A is a constant, and $E_{g}$ is the bandgap of the ZIS samples. The ZIS is a direct band gap semiconductor, so the index is 2.

(3) $$\frac{K}{S}=\frac{{\left(1-R_{\infty }\right)}^{2}}{2R_{\infty }}=F_{R}$$

where *K* is the absorption coefficient at a specific wavelength, *S* is the scattering coefficient at a specific wavelength, and *R*_∞_ is the reflectance of the ZIS sample.

(4) $${\left(F_{R}h\nu \right)}^{2}=B\left(h\nu -E_{g}\right)$$

where $F_{R}$ can be obtained from Equation (3), hν is the photon energy, *B* is a constant, and $E_{g}$ is the bandgap, as mentioned previously.

### 2.4. Photocatalysis of CO_2_ Reduction Reaction (CO_2_RR)

The photocatalytic reaction of carbon dioxide was conducted as follows in Figure S1. Initially, 10 mg of the ZIS sample powder was uniformly spread within a 180 mL glass reactor. Subsequently, 3 mL of a 10% aqueous solution of triethanolamine (TEOA) was added to the reactor to facilitate the reaction. Carbon dioxide gas (99.99%) was introduced into the reactor by passing it through a gas washing bottle filled with deionized water, allowing for the entrainment of moisture along with the carbon dioxide gas. After continuous aeration for 50 min, the glass reactor was sealed to commence the CO_2_RR under simulated sunlight conditions using a solar simulator (XES-502S, San-Ei Electric, Osaka, Japan) with an intensity of 100 mW cm^−2^.

Following the photocatalytic reaction, the resulting gaseous products were analyzed using gas chromatography (GC, TRACE™ 1600 Series) equipped with a fused-silica capillary column (Supel-Q™, 30 m × 0.53 mm) and a TG BOND column (Msieve 5A, 30 m × 0.53 mm) to determine the presence and quantity of carbon monoxide and methane. The gaseous product CO was analyzed at 70 °C for 8 min using helium as a carrier gas at a flow rate of 9 mL min^−1^ and a TG BOND column coupled with a thermal conductivity detector (TCD). The gaseous product CH_4_ was analyzed at 70 °C for 8 min using argon as a carrier gas at a flow rate of 20 mL min^−1^ and a Supel-Q column coupled with a flame ionization detector (FID). Calibration curves were utilized to estimate the yield of these photocatalytic products accurately.

## 3. Results and Discussion

### 3.1. The Morphology, Crystal Structure, and Chemical State of Zinc Indium Sulfide (ZIS) Nanocrystals

**Figure 1:**
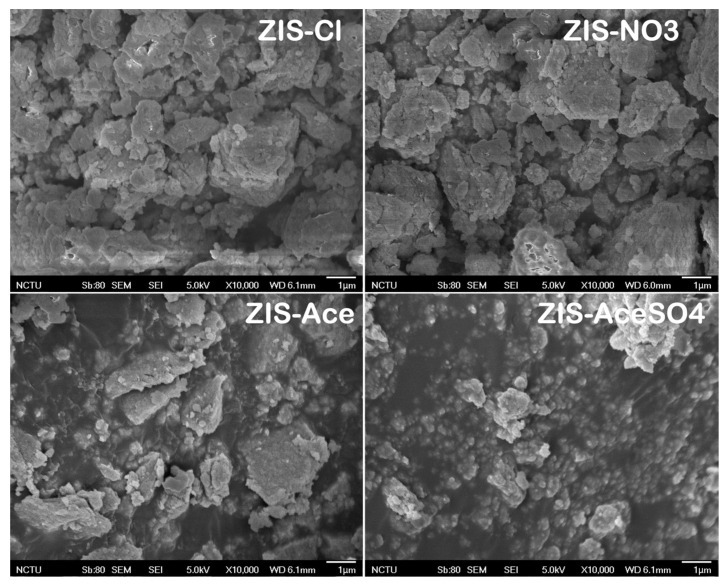
Scanning electron microscopy (SEM) images showing the crystal morphologies of zinc indium sulfide (ZIS) semiconductor materials studied in this work. The crystal sizes varied between the different variants: ZIS-Cl and ZIS-NO_3_ predominantly exhibited micron-sized crystals (larger than one micron), while ZIS-Ace and ZIS-AceSO_4_ displayed a higher prevalence of nanoscale crystals (smaller than one micron).

The hydrothermal synthesis of the zinc indium sulfide (ZnIn_2_S_4_) semiconductor materials yielded four distinct products: ZIS-Cl (containing chloride salts), ZIS-NO_3_ (containing nitrate salts), ZIS-Ace (containing acetate salts), and ZIS-AceSO_4_ (containing both acetate and sulfate salts). The morphological characteristics and crystal structures of these materials were compared, as shown in Figure 1. In Figure 1, the SEM images revealed that all four ZIS materials exhibited irregular nanostructures, with nanoscale crystals forming aggregated structures. Specifically, the ZIS-Cl and ZIS-NO_3_ samples exhibited larger, micron-sized aggregated crystals, whereas ZIS-Ace and ZIS-AceSO_4_ displayed smaller, nanometer-sized grain structures. This feature underscored the significant influence of different precursors on the morphology and size of zinc indium sulfide crystals. To expand on this, the choice of precursor not only affected the grain size and aggregation state of the resulting crystals but also their potential applications and properties. For instance, the smaller nanometer-sized grains in ZIS-Ace and ZIS-AceSO_4_ could be beneficial for applications demanding high surface reactivity and enhanced catalytic properties due to their higher surface-to-volume ratios.

In addition, the observed morphological variations could be attributed to the different ionic interactions and solubility properties of the precursor salts used in the hydrothermal synthesis process. Chloride and nitrate ions, for instance, tended to form more stable and larger crystalline structures under hydrothermal conditions, leading to the observed micron-sized aggregates. On the other hand, acetate and sulfate ions might have facilitated the formation of smaller, more dispersed nanocrystals due to their differing solubility dynamics and interaction strengths with the zinc indium sulfide matrix.

Additionally, the surface elemental composition of the four samples was analyzed using energy-dispersive X-ray spectroscopy (EDS) in combination with field emission scanning electron microscopy (FESEM); the corresponding results are shown in Table S1. Notably, while the ZIS-Cl sample exhibited the ideal Zn:In:S~1:2:4 elemental ratio, the other samples showed zinc and indium atomic ratios of less than 1 and 2, respectively [47]. This indicated that despite all four ZIS samples being synthesized with a precursor molar ratio of Zn:In:S = 1:2:4, the different anionic salt precursors led to preferential compositional ratios, resulting in the formation of zinc and indium vacancy defects during the hydrothermal process.

To elaborate, the deviation from the ideal stoichiometry in the ZIS-NO_3_, ZIS-Ace, and ZIS-AceSO_4_ samples suggested that the type of anionic precursor used significantly influenced the incorporation efficiency of zinc and indium atoms into the crystal lattice. The presence of vacancy defects could have profound effects on the electronic and optical properties of the materials. For instance, zinc and indium vacancies could introduce localized energy states within the bandgap, potentially enhancing the material’s ability to participate in charge carrier processes such as charge separation and photocatalysis.

The formation of these defects could be attributed to several factors, including the differential solubility of the precursors, the stability of the intermediate species during synthesis, and the kinetics of crystal growth under hydrothermal conditions. Chloride ions, for example, might facilitate a more complete incorporation of zinc and indium due to their smaller ionic radius and higher solubility, leading to the ideal stoichiometric ratio observed in the ZIS-Cl sample. In contrast, nitrate, acetate, and sulfate ions might disrupt this balance, leading to the observed deficiency in zinc and indium content.

**Figure 2:**
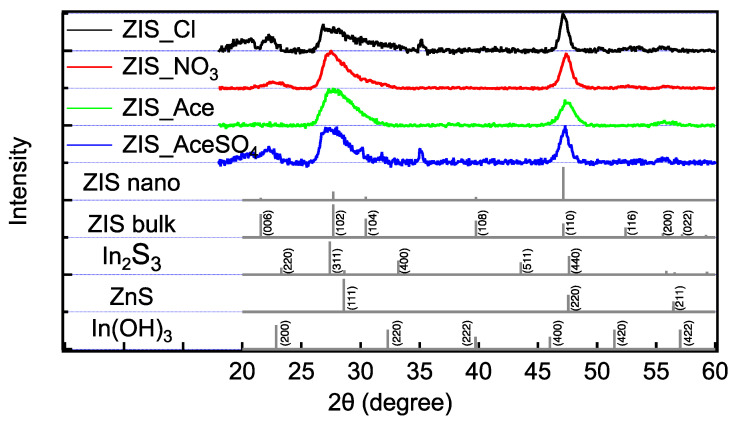
X-ray powder diffraction patterns of zinc indium sulfide materials studied in this work. From top to bottom, the patterns correspond to ZIS-Cl (black line), ZIS-NO_3_ (red line), ZIS-Ace (green line), ZIS-AceSO_4_ (blue line), and references from the literature (gray lines). The characteristic peaks of ZIS were observed at 20°, 22°, 27°, and 47°.

Further analysis of the crystal crystallinity of the aggregated nanocrystals in zinc indium sulfide powders was conducted using X-ray diffraction (XRD), as depicted in Figure 2. The XRD patterns revealed notable diffraction peaks at 20°, 22°, 27°, and 47° for each of the four samples. Among these peaks, those at 27° and 47° were particularly prominent [47,48,49]. These diffraction peaks corresponded to specific crystallographic planes and indicated the presence of well-defined crystalline phases in the synthesized zinc indium sulfide samples. The peak at 27° was typically associated with the (102) plane of the bulk-ZIS (micron-sized) structure, while the peak at 47° corresponded to the (110) plane of the nano-ZIS (nano-sized) structure. The prominence of these peaks suggested that the samples exhibited good crystallinity and that the crystal structure predominantly adopted the zinc indium sulfide phase [50,51]. The observed diffraction patterns also provided insights into the size and orientation of the crystalline domains within the aggregated nanocrystals. The relative intensities and positions of the peaks could further inform about the degree of crystallinity, the grain size distribution, and the potential presence of any secondary phases or defects within the zinc indium sulfide structure.

The ZIS of these four different anions all had obvious diffraction peaks at 27° and 47°. This suggested that the four ZIS materials synthesized via hydrothermal methods exhibited characteristics of both bulk and nanomaterials. Notably, ZIS-Ace differed from the other samples in that it lacked prominent peaks at 20° and 22°, which are characteristic of ZIS-bulk materials. Additionally, In(OH)_3_ was not observed in all the hydrothermal synthesized samples, indicating that In^2+^ was not oxidized. In addition, ZnS and In_2_S_3_ showed some overlapping peaks with the ZIS signals, but no additional distinct features were observed outside these positions. Therefore, the ZIS synthesized via the hydrothermal method primarily consisted of ZnIn_2_S_4_ rather than other by-products.

Further analysis using the Scherrer equation [52] was applied to determine the crystal sizes of the ZIS samples, with the results presented in Table S2. Interestingly, ZIS-Ace demonstrated the smallest crystal size at 4.8 nm. In combining these XRD findings with the SEM analysis, it was evident that ZIS-Ace possessed a morphology and structure rich in nano-sized features compared to the other three samples. Therefore, for applications requiring a higher proportion of nano-sized zinc indium sulfide crystals, using acetate salts as precursors in the synthesis process might be preferred. This choice influenced not only the crystal size but also potentially enhanced specific properties such as the surface area, reactivity, and performance in various technological applications like photocatalysis.

On the other hand, the chemical states of zinc indium sulfide (ZIS) materials with different anionic salts were examined using X-ray photoelectron spectroscopy (XPS), which measured the binding energies of elements on the material’s surface, as depicted in Figure S3. The binding energies of the four ZIS materials were compared across the entire scan range (−10 eV to 1350 eV), focusing specifically on zinc atoms (1010 eV to 1060 eV), indium atoms (435 eV to 462 eV), and sulfur atoms (157 eV to 175 eV). The surface chemical bonding of zinc, indium, and sulfur elements in the four ZIS samples exhibited remarkable similarity. This suggested that the different anions used did not significantly influence the formation of ZnIn_2_S_4_, nor did they affect the oxidation states of the metals or the chemical bonding within the materials. No significant shifts in binding energies were observed among the four samples, indicating consistent chemical environments and very similar compositions across all the ZIS samples. These findings implied that while the choice of anionic precursor salts influenced the morphology, defects, and structural aspects of the ZIS crystals, such as the size and aggregation state as observed in previous analyses, it did not substantially alter the chemical bonding characteristics of the materials. For example, the metal oxide or by-products could not be detected. This consistency in chemical bonding further supported the reproducibility and reliability of the hydrothermal synthesis method for producing zinc indium sulfide with consistent chemical structures, regardless of the specific precursor used.

### 3.2. The Bandgap Estimation of Zinc Indium Sulfide (ZIS) Nanocrystals

From the surface characterization and elemental composition, which were analyzed using SEM, XRD, and XPS, no significant differences were observed among the four ZIS nanocrystals. However, variations in the colors of the powder samples were noted, as shown in Figure S4. Specifically, ZIS-Cl and ZIS-NO_3_ displayed an earthy yellow color, while ZIS-Ace appeared darker yellow. In contrast, ZIS-AceSO_4_ was noticeably lighter and slightly whitish compared to the other three ZIS powders. This color variation suggested differences in the optical properties of the ZIS materials, potentially indicating variations in their bandgaps. To explore this further, the diffuse reflectance spectra of the powder samples were measured using an integrating sphere combined with a UV-Vis-NIR spectrophotometer. Since the sample thickness prevented light from transmitting through it, the transmittance could be ignored. The absorbance (A) of the samples could be calculated using the following equations:

A + T + R + S = 1, T = 0(5)

A = 1 − (R + S), A = 1 − %R(6)

where T is the transmittance, R is the reflectance, S is the scattering, and %R is the proportion of reflectance from the UV-Vis spectrophotometer.

**Figure 3:**
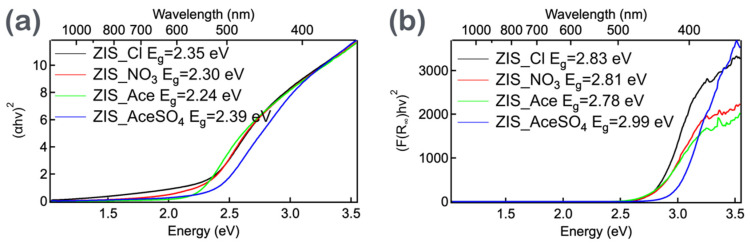
(**a**) The bandgap of ZIS powder samples determined using Tauc plot analysis, where ${\left(\alpha h\nu \right)}^{2}$ is plotted against hν and linear regression is performed. The bandgaps for ZIS-Cl, ZIS-NO_3_, ZIS-Ace, and ZIS-AceSO_4_ were determined to be 2.35 eV, 2.30 eV, 2.24 eV, and 2.39 eV, respectively. (**b**) The bandgap of ZIS powder samples, corrected using the Kubelka–Munk equation and determined by plotting (F_R_hν)^2^ against hν and performing linear regression. The bandgaps for ZIS-Cl, ZIS-NO_3_, ZIS-Ace, and ZIS-AceSO_4_ were determined to be 2.83 eV, 2.81 eV, 2.78 eV, and 2.99 eV, respectively.

Using these equations, the absorbance spectra of the ZIS samples were plotted (Figure S5a), and the bandgaps (E_g_) were estimated through Tauc plot analysis (Figure 3a). The absorption spectra revealed an increase in absorbance from 600 to 1200 nm, indicating significant surface roughness and scattering phenomena in the powder samples. Consequently, in the Tauc plot, there was a background increase in the range of 1.0 to 2.0 eV. By linearly fitting the intersections of the lines between 1.0 to 2.0 eV and 2.2 to 2.5 eV, the bandgaps for ZIS-Cl (Eg = 2.35 eV), ZIS-NO_3_ (Eg = 2.30 eV), ZIS-Ace (Eg = 2.24 eV), and ZIS-AceSO_4_ (Eg = 2.39 eV) were determined. Although the aforementioned results indicated that different anionic precursors in the hydrothermal process influenced the optical properties of the ZIS materials, such as the bandgaps, a crucial issue remained unresolved: if a sample exhibits absorption in the visible to near-infrared region, its color should appear black. However, the ZIS powders were all yellowish and somewhat white, suggesting that the absorption wavelengths fell within the blue light region rather than across the entire visible spectrum. Despite the fact that the bandgaps of the four ZIS samples were close to most of the values in the literature (2.24~2.39 eV), it was essential to use an appropriate mathematical model to accurately calculate the actual bandgaps of the samples.

Therefore, the Kubelka–Munk (KM) equation was commonly used to describe the propagation of light in non-homogeneous media. This mathematical model, jointly proposed by Kubelka and Munk, assumed that when the sample thickness tended to infinity, the reflectance value was *R*_∞_. When the sample thickness was considered opaque, the measured reflectance, R, could be approximated to *R*_∞_. The equation is given by:

(7) $$R_{\infty }=1+\frac{K}{S}-\sqrt{\frac{K}{S}\left(2+\frac{K}{S}\right)}$$

where *K* is the absorption coefficient at a specific wavelength and *S* is the scattering coefficient at a specific wavelength. After simplification, Equation (3) can be obtained.

The plotting of F_R_ against the wavelength and (*F_R_hν*)^2^ against *hν* could be used to estimate the bandgap of the sample, as shown in Figure 3b and Figure S5b, respectively. After correction using the Kubelka–Munk equation, the original bandgap range of 2.24 to 2.39 eV for the ZIS materials was extended to 2.78 to 2.99 eV. Additionally, considering that the ZIS powder itself appeared yellow, with ZIS-AceSO_4_ leaning toward yellow-white, it was reasonable for the bandgap to fall within the blue light region. Through the plotting and calculation of bandgaps using the aforementioned equations, it was evident that the choice of mathematical model could lead to significant differences in estimating the bandgaps for these materials. This highlighted the importance of using appropriate models for accurate bandgap determination, reflecting the true optical properties and behavior of the materials.

### 3.3. The Energy Level of Zinc Indium Sulfide (ZIS) Nanocrystals

After measuring the different bandgaps of the four ZIS samples, further insights into the energy levels of the valence band (VB) and conduction band (CB) of these semiconductor materials could be obtained using ultraviolet photoelectron spectroscopy (UPS). This provided valuable information for the subsequent design of photocatalytic applications, including the selection of reactants, the heterojunction designed, and the generation of products, which could be helpful for experimental design.

In the UPS measurements, the Fermi level (E_fermi, Au_) is indicated by the vertical dashed line in Figure S6, positioned at −0.05 eV. From the UPS measurements, the work function (Φ) and the maximum value of the valence band maximum (VBM) of the materials could be obtained. Based on the linear regression results, the work function (Φ) values of the four ZIS materials, ZIS-Cl, ZIS-NO_3_, ZIS-Ace, and ZIS-AceSO_4_, were determined to be −5.55 eV, −5.58 eV, −5.57 eV, and −5.56 eV, respectively. The corresponding VBM values were determined to be −6.56 eV, −6.65 eV, −6.72 eV, and −6.64 eV, respectively. The differences in the work function values were negligible, whereas there was a difference of approximately 0.1 eV in the VBM values.

**Figure 4:**
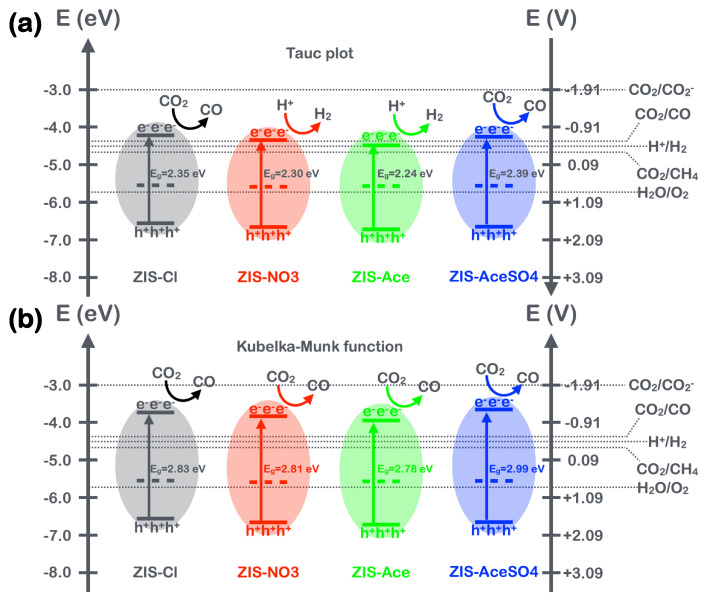
(**a**) The band diagram obtained from the band gaps estimated by the Tauc plot and VBM through UPS measurements. (**b**) The band diagram obtained from the band gaps estimated using the Kubelka–Munk function and VBM through UPS measurements.

Integrating these results with the estimated bandgaps from the absorption spectra allowed the construction of the energy band diagram of the ZIS semiconductor materials. First, the band diagram obtained from the bandgaps estimated by the Tauc plot is shown in Figure 4a. It could be inferred from this diagram that the conduction band minimum (CBM) of the ZIS samples was near the energy levels relevant for the carbon dioxide reduction reaction (CO_2_RR) and the hydrogen evolution reaction (HER). When the energy band diagram was corrected using the Kubelka–Munk model, as shown in Figure 4b, the CBM was increased by approximately 0.5 eV. This indicated that despite the presence of defects in the ZIS materials, the defect energy levels were still sufficient to initiate the photocatalytic process, mitigating the risk of material incapacitation due to energy level mismatch.

Another intriguing aspect was that when ZIS forms heterojunctions with other semiconductors, the energy levels often undergo changes, resulting in band bending. According to the results shown in Figure 4a, band bending might have caused the reduction in the CBM from the CO_2_RR and HER regions to below the catalytic energy level, leading to the catalyst being inactive. However, based on the Kubelka–Munk modification results shown in Figure 4b, even with band bending and rearrangement, the reduced energy levels were still higher than those required for CO_2_RR and HER. This significant discrepancy (~0.5 eV) between the bandgaps predicted by the Tauc plot model and the Kubelka–Munk model indicated that the CBM predicted by the Tauc plot model would face difficulty in initiating the CO_2_RR to generate CO. We discuss this issue in the following sections to explore the implications of this discrepancy for photocatalytic performance and material design. 

### 3.4. The CO_2_ Reduction Efficiency of Zinc Indium Sulfide (ZIS) Nanocrystals

**Figure 5:**
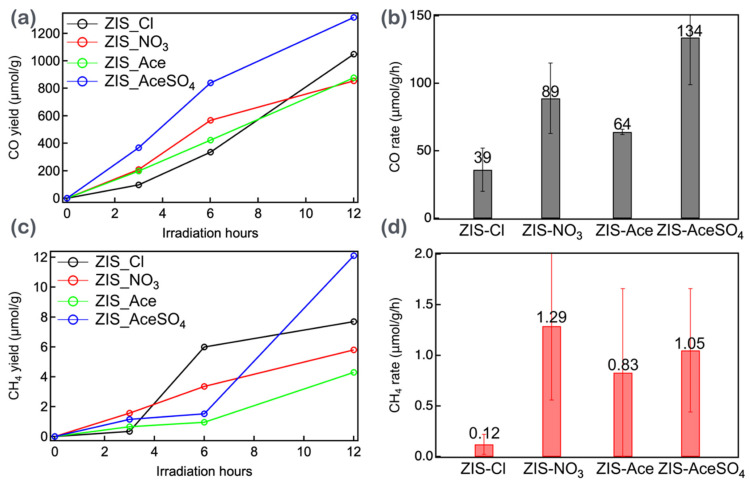
(**a**) The CO production yields as a function of irradiation time. (**b**) The CH_4_ production yields as a function of irradiation time. (**c**) The CO production rate estimated from (**a**) for the first three hours. (**d**) The CH_4_ production rate estimated from (**b**) for the first three hours.

Finally, these four types of ZIS powders were subjected to photocatalytic CO_2_ reduction experiments, with the results shown in Figure 5. The photocatalytic CO_2_ reduction of the ZIS samples produced two types of gaseous products: carbon monoxide (CO) and methane (CH_4_). CO was found to be the major photoproduct, while CH_4_ was the minor product. Upon illuminating the samples for 3, 6, and 12 h, it was revealed that the CO production rates were highest for ZIS-AceSO_4_ (~1300 μmol g^−1^ for 12 h). Similarly, the methane production rates were also highest for ZIS-AceSO_4_ (~12 μmol g^−1^ for 12 h). Interestingly, examining the photocatalytic efficiencies revealed that ZIS-AceSO_4_, with the largest bandgap, provided the best CO yield compared to the others. The CO production rate trend, as shown in Figure 5b, was ZIS-AceSO_4_ (134 μmol g^−1^h^−1^) > ZIS-NO_3_ (89 μmol g^−1^h^−1^) > ZIS-Ace (64 μmol g^−1^h^−1^) > ZIS-Cl (39 μmol g^−1^h^−1^). This trend in CO production rates was consistent with the zinc and indium vacancy defects determined from the EDS analysis (Table S1). However, for the small amounts of CH_4_ production, the rates showed a trend of ZIS-NO_3_ (1.29 μm g^−1^h^−1^) > ZIS-AceSO_4_ (1.05 μm g^−1^h^−1^) > ZIS-Ace (0.83 μm g^−1^h^−1^) > ZIS-Cl (0.12 μm g^−1^h^−1^), but the uncertainties were quite large, as shown in Figure 5c. At the irradiation time of 12 h, ZIS-AceSO_4_ still yielded the highest CH_4_ among the samples. 

Furthermore, oxidation reaction tests for the optimized ZIS-AceSO_4_, which exhibited the highest CO production rate, were conducted under three conditions: (i) anhydrous (no water) solution, (ii) hydrated solution (with water), and (iii) aqueous solution containing 10% TEOA. The results, shown in Figure S7, indicated that regardless of the presence of water, CO_2_RR was very inefficient, yielding only 10 μmol g^−1^ and 19 μmol g^−1^ of CO production under anhydrous and hydrated conditions in 12 h, respectively. However, upon adding sacrificial agents like TEOA, the CO production yield increased dramatically to 1764 μmol g^−1^ in 12 h, indicating that TEOA played a crucial role in enhancing CO production with ZIS-AceSO_4_. It is important to note that the production of CO and CH_4_ was negligible for the TEOA aqueous solution in the absence of CO_2_. This suggested that although the reduction potential energy level was sufficient to convert CO_2_ to CO/CH_4_, the oxidation half-reaction was a bottleneck that could be overcome by using a sacrificial agent like TEOA.

The possible mechanistic pathways for CO_2_RR using ZnIn_2_S_4_ are as follows: First, under visible light irradiation, ZnIn_2_S_4_ absorbs photons, exciting electrons from the valence band to the conduction band, generating electron-hole pairs. Second, effective charge separation is crucial to prevent the recombination of photogenerated electron-hole pairs. ZnIn_2_S_4_, especially with defect formation, facilitates this separation and transfer, enhancing photocatalytic activity. Third, CO_2_ molecules are adsorbed onto the surface of the ZnIn_2_S_4_ photocatalyst. The presence of structural defects in ZnIn_2_S_4_ increases its CO_2_ adsorption capacity. The photogenerated electrons reduce the adsorbed CO_2_ molecules through several intermediate steps. In situ FT-IR experiments indicated the presence of a COO* intermediate pathway, leading to the formation of CO as the primary reduction product [53]. Finally, in comparing the ZIS-AceSO_4_ synthesized under the hydrothermal method in this study with previously reported ZIS samples, it could be observed (Table S3) that most research teams used chloride or nitrate salts as precursors for ZIS synthesis. Similarly, in this study, ZIS-Cl and ZIS-NO_3_ were synthesized using the same precursors and subjected to photocatalytic reactions. However, the ZIS-AceSO_4_ utilized in this research demonstrated higher efficiency in CO_2_RR, highlighting the significance of investigating different anionic environments. Additionally, when comparing ZIS-AceSO_4_ with other semiconductor materials with CO_2_RR potential, as shown in Table S4, it became evident that ZIS-AceSO_4_ exhibited superior CO_2_RR performance. The advantages of ZIS semiconductor materials, such as not requiring the use of noble metals, having a simple synthesis method, and possessing low toxicity, make ZIS an increasingly attractive candidate for future applications as a heterojunction material. This research underscored the importance of exploring alternative anionic precursors in the hydrothermal synthesis of ZIS materials. The findings suggested that using acetate and sulfate salts could lead to significant enhancements in photocatalytic efficiency. This opens new avenues for optimizing the photocatalytic properties of ZIS materials and other semiconductors through careful selection of precursor materials, thereby advancing the development of efficient and sustainable photocatalytic systems.

## 4. Conclusions

**Figure 6:**
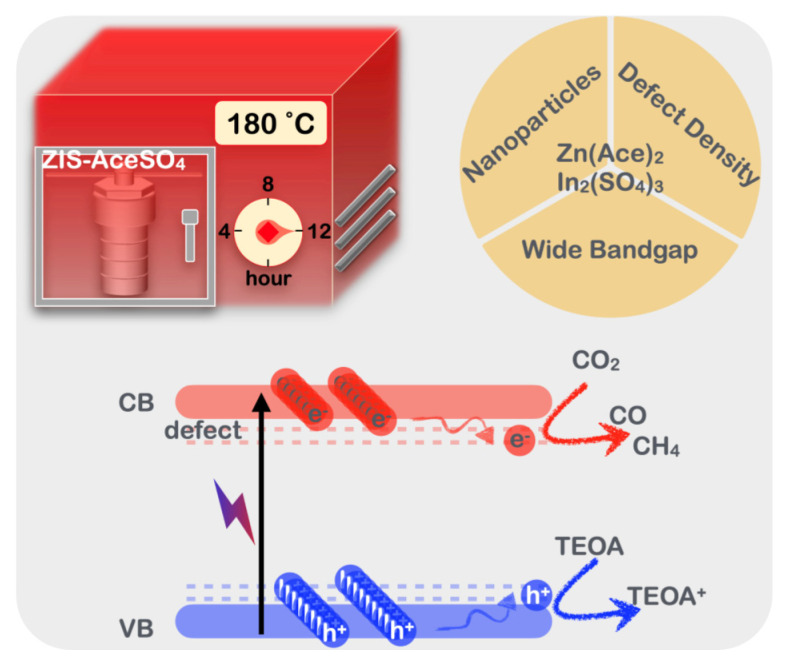
The schematics of ZIS nanoparticle synthesis, key processes, and photocatalytic CO_2_ reduction.

In our investigation, we synthesized and characterized zinc indium sulfide (ZIS) semiconductor materials via the hydrothermal method using four different anionic salt precursors, ZIS-Cl, ZIS-NO_3_, ZIS-Ace, and ZIS-AceSO_4_, to explore efficient photocatalysts for CO_2_ reduction reactions. CO_2_ reduction reactions were conducted in an aqueous solution containing 10% triethanolamine under one-sun irradiation. We observed that the CO production rates followed the trend ZIS-AceSO_4_ (134 μmol g^−1^h^−1^) > ZIS-NO_3_ (89 μmol g^−1^h^−1^) > ZIS-Ace (64 μmol g^−1^h^−1^) > ZIS-Cl (39 μmol g^−1^h^−1^), which is consistent with the zinc and indium vacancy defects confirmed by energy dispersive X-ray spectroscopy (EDS) measurements. A significant discrepancy was observed in the bandgap estimation through the Tauc plot and Kubelka–Munk analysis, underscoring the importance of selecting the appropriate mathematical model. The valence band maximum (VBM) energy levels were determined through ultraviolet photoelectron spectroscopy (UPS), while the conduction band minimum (CBM) energy levels predicted by the Kubelka–Munk model were approximately 0.5 eV higher than those predicted by the Tauc plot model. Thus, the former model is deemed preferable for more efficient CO_2_ reduction reactions to occur. Our study highlights the influence of anionic salt precursors on the preparation of ZIS nanomaterials, particularly in creating more zinc and indium vacancy defects to enhance product yields in photocatalytic CO_2_ reduction reactions. These findings hold significant implications for industrial applications of photocatalytic CO_2_ reduction. By selecting appropriate precursors and mathematical models, we can achieve more efficient CO_2_ conversion, contributing to climate change mitigation and the development of renewable energy technologies. A schematic demonstration for the present study is shown in Figure 6.

## Data Availability

Data is contained within the article and Supplementary Material.

## Supplementary Materials

The following supporting information can be downloaded at https://www.mdpi.com/article/10.3390/nano14141231/s1, refs. [22,35,43,44,54,55,56,57,58,59,60,61] is cited in the Supplementary Materials.
