# Supplementary material for: Photocatalytic CO2 Reduction Using Zinc Indium Sulfide Aggregated Nanostructures Fabricated under Four Anionic Conditions
Source: Nanomaterials (Basel). 2024 Jul 20;14(14):1231. doi: 10.3390/nano14141231 (PMC11280036; doi:10.3390/nano14141231)
Supplement: Supplementary file 1 [file nanomaterials-14-01231-s001.zip › nanomaterials-3115133-supplementary.pdf]

Supporting information

Photocatalytic CO_2 Reduction Using Zinc Indium Sulfide Aggregated Nanostructures Fabricated Under Four Ionic Conditions

I-Hua Tsai[1], *and Eric Wei-Guang Diau*[1,*]

[1] Department of Applied Chemistry and Institute of Molecular Science, National Yang Ming Chiao Tung University, 1001 Ta-Hseuh Rd., Hsinchu 300093, Taiwan.

[2] Center for Emergent Functional Matter Science, National Yang Ming Chiao Tung University, 1001 Ta-Hseuh Rd., Hsinchu 300093, Taiwan.

* Correspondence: diau@nycu.edu.tw (E.W.-G.D.)

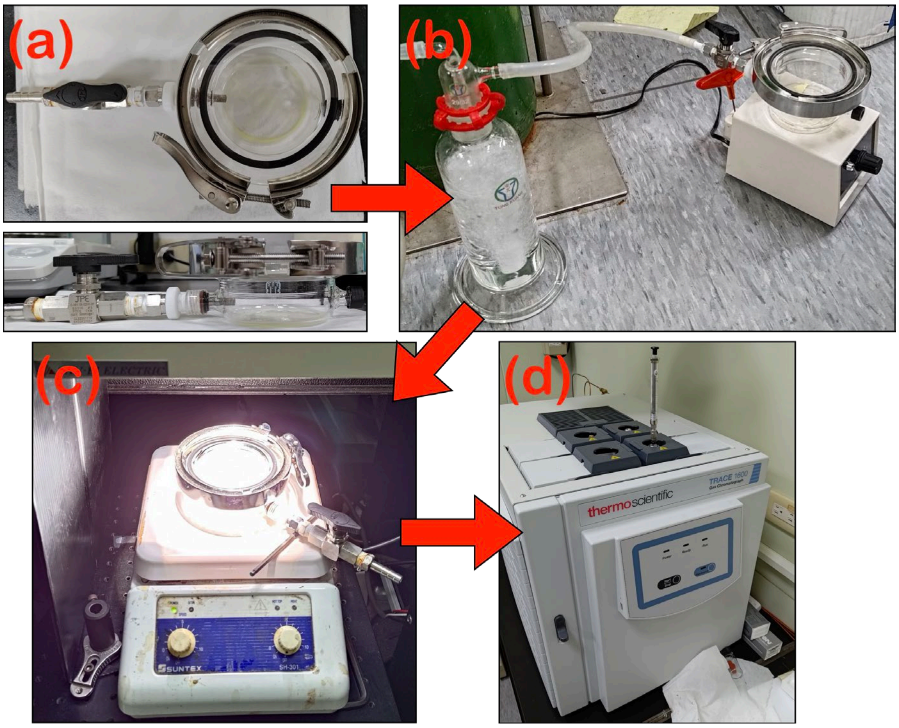

Figure S1. The flowchart of the photocatalytic CO_2 reduction reaction process. (a) 10 mg ZIS powder and 3 mL 10% TEOA solution spread within a 180 mL glass reactor. (b) CO_2 (99.99%) was introduced into the reactor by passing it through a gas washing bottle filled with deionized water for 50 minutes. (c) The reactor was illuminated in the solar simulator for 12 hours of photoreaction. (d) The resulting gaseous products were analyzed using gas chromatography.

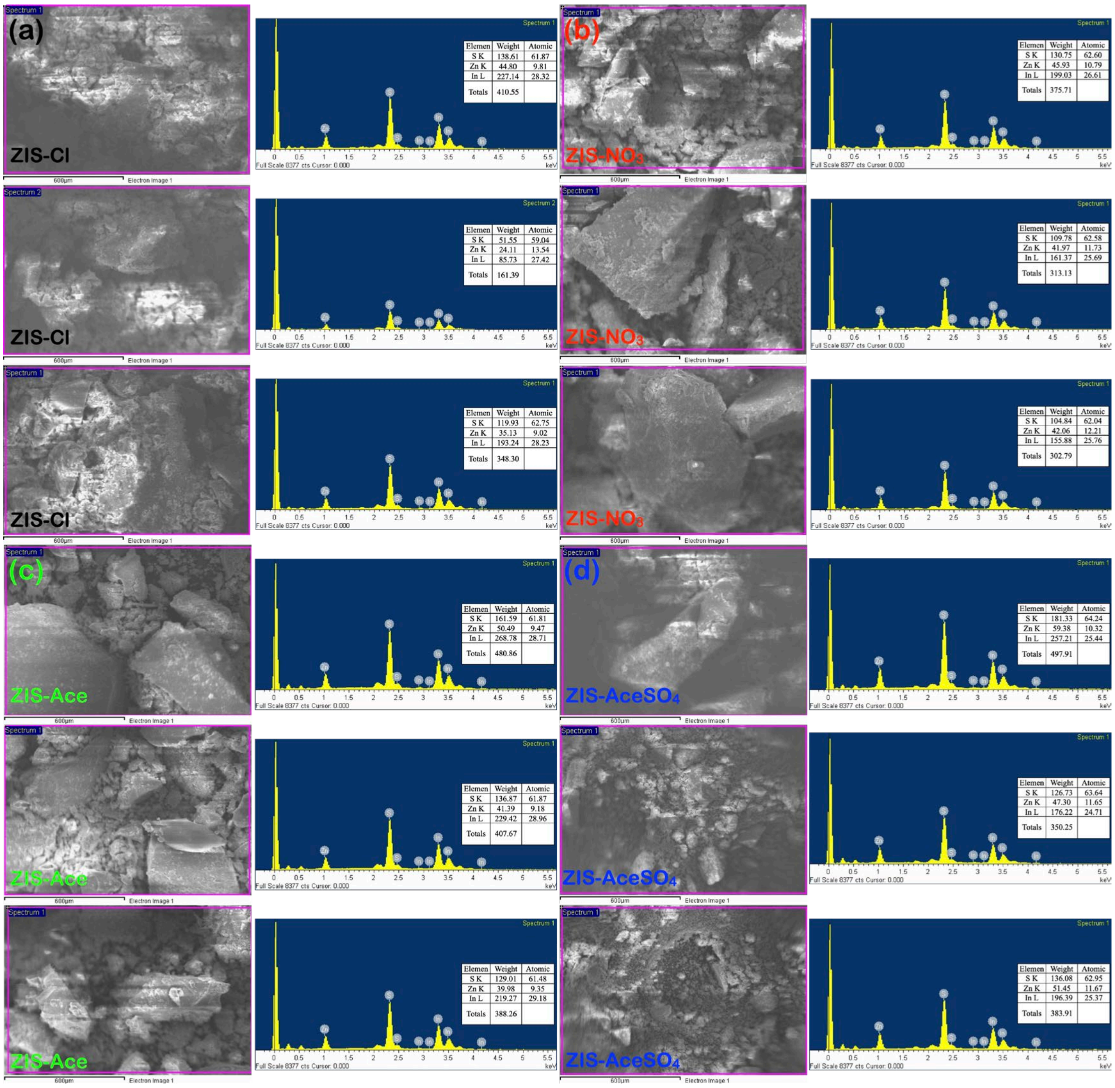

Figure S2. The EDS images of ZIS-Cl, ZIS-NO_3, ZIS-Ace, and ZIS-AceSO_4 are shown, with the atomic ratios also presented in the figures. The average atomic ratios are listed in Table S1.

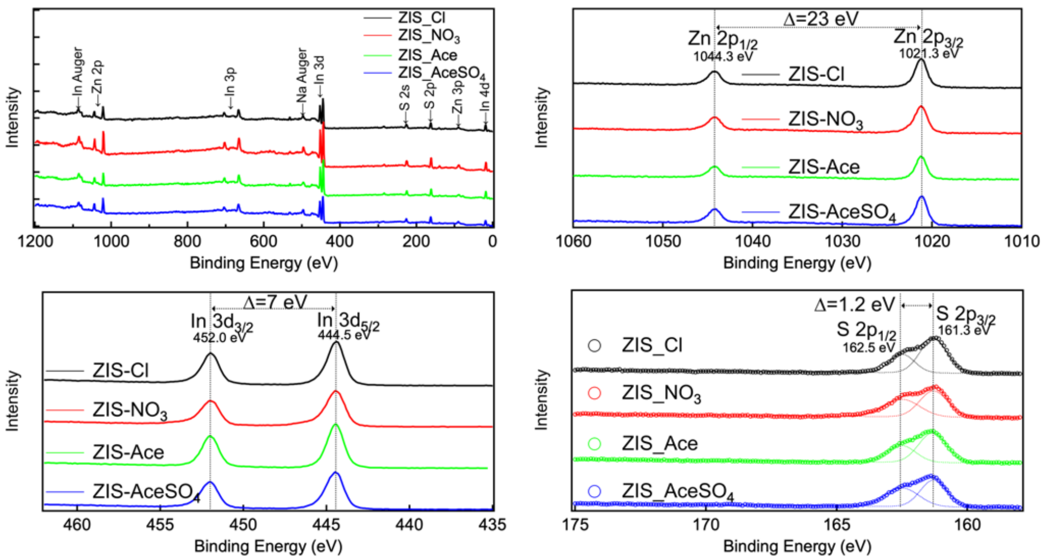

Figure S3. X-ray photoelectron spectra of zinc indium sulfide materials are presented. The spectra are arranged sequentially for ZIS-Cl (black line), ZIS-NO_3 (red line), ZIS-Ace (green line), and ZIS-AceSO_4 (blue line). The binding energies for Zn $2p_{3/2}$ and Zn $2p_{1/2}$ are 1021.3 eV and 1044.3 eV, respectively. For In $3d_{5/2}$ and In $3d_{3/2}$, the binding energies are 444.5 eV and 452.0 eV, respectively. The binding energies for S $2p_{3/2}$ and S $2p_{1/2}$ are 161.3 eV and 162.5 eV, respectively.

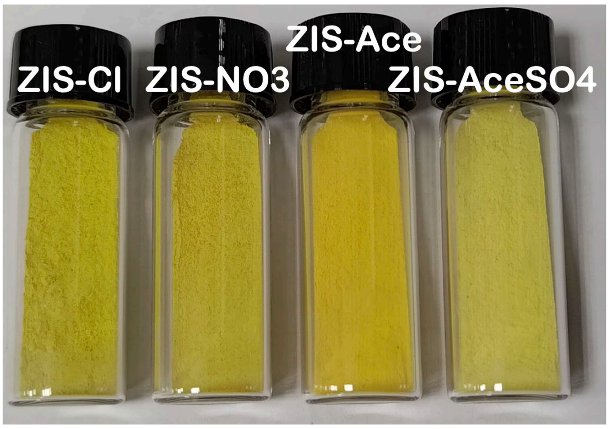

Figure S4. The colors of the four ZIS powders under sunlight illumination are presented from left to right as follows: ZIS-Cl, ZIS-NO_3, ZIS-Ace, and ZIS-AceSO_4. ZIS-Cl and ZIS-NO_3 exhibited an earthy yellow color, while ZIS-Ace appeared as a darker yellow. And ZIS-AceSO_4 appeared lighter and slightly whitish.

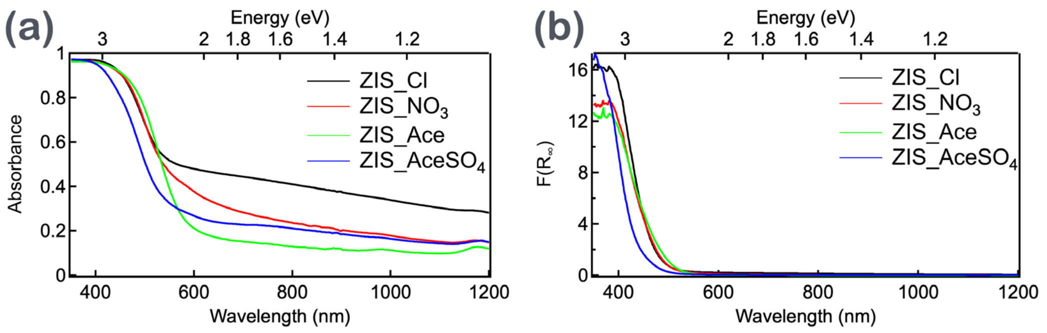

Figure S5. The absorption spectra of ZIS powder samples were obtained. (a) The diffuse reflectance spectra were measured using an integrating sphere to calculate the reflectance. Then, the absorbance was calculated using the Equation 6, A=1−%R, where A represents absorbance and %R is the reflectance. (b) The Kubelka-Munk equation corrected ZIS powder sample is plotted as F_R against the wavelength using the Equation 3, $\frac{K}{S} = \frac{(1-R_\infty)^2}{2R_\infty} = F_R$.

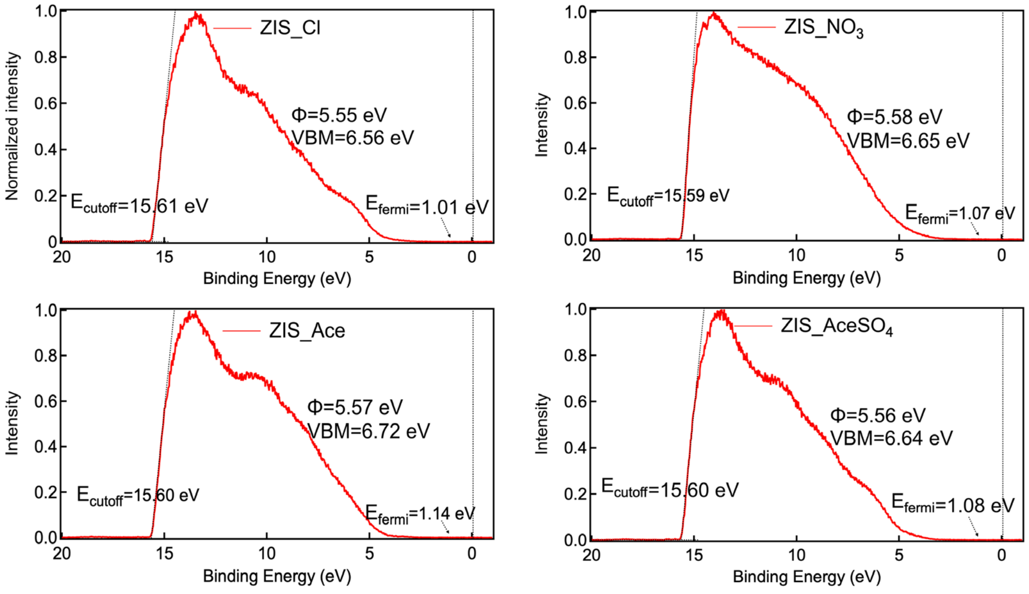

Figure S6. The ultraviolet photoelectron spectra (UPS) of four ZIS powder samples as indicated. Linear regression was employed to fit the low-energy region and the high-energy cutoff region, allowing the determination of the cutoff energy (E_{cutoff}) and the Fermi level (E_{fermi}). Given, $E_{fermi, Au}$=-0.05 eV, the work function (Φ) values for the four ZIS materials, ZIS-Cl, ZIS-NO_3, ZIS-Ace, and ZIS-AceSO_4, were found to be 5.55 eV, 5.58 eV, 5.57 eV, and 5.56 eV, respectively. Additionally, the values of the maximum of the valence band (VBM) were determined to be 6.56 eV, 6.65 eV, 6.72 eV, and 6.64 eV, respectively, for the four materials.

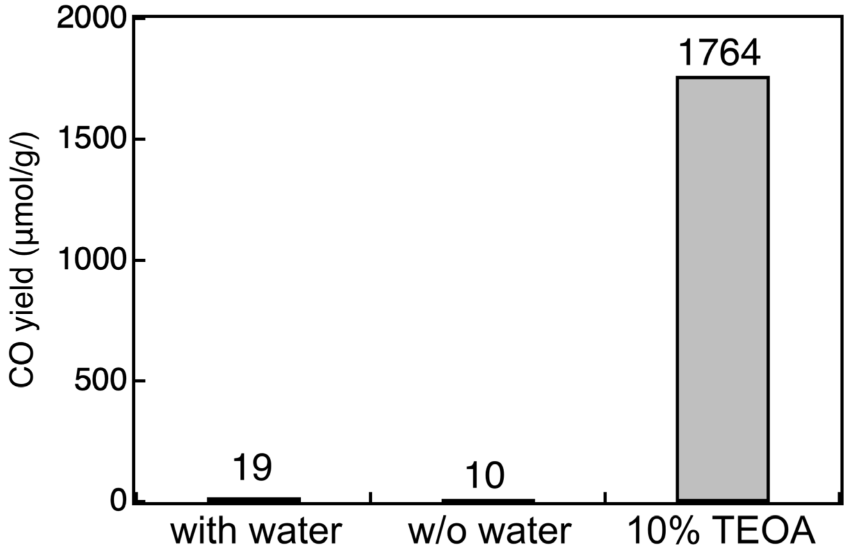

Figure S7. CO production yields of ZIS-AceSO4 with photocatalytic reactions for 12 hours under three different conditions: dry condition (without water), wet condition (with water), and in a 10% TEOA aqueous solution.

Table S1. Surface elemental analysis of zinc indium sulfide. The surface composition of zinc, indium, and sulfur atoms in each sample was analyzed using energy-dispersive X-ray spectroscopy (EDS).

**Samples**	**Zn**	**In**	**S**
ZIS-Cl	0.9 (13.54%)	1.9 (27.42%)	4 (59.04%)
ZIS-NO_3	0.8 (12.21%)	1.7 (25.76%)	4 (62.04%)
ZIS-Ace	0.6 (9.47%)	1.9 (28.71%)	4 (61.81%)
ZIS-AceSO_4	0.7 (11.67%)	1.6 (25.37%)	4 (62.95%)

Table S2. Crystalline size of zinc indium sulfide. The Scherrer equation $D = \frac{K\lambda}{\beta \cos \theta}$, where K is shape factor (K=0.94 for spherical crystalline), λ is the wavelength of x-ray beam (λ=0.15406 nm), θ is diffraction angle, and β is the full width at half maximum.

	2θ (°)	Reflection Planes	FWHM (radians)	Crystalline size (nm)	Average size (nm)
ZIS-Cl	27.5	(102)	0.033	4.54	5.9
	30.0	(104)	0.076	1.97	
	47.2	(110)	0.014	11.33	
ZIS-NO_3	27.5	(102)	0.027	5.52	5.5
	29.2	(104)	0.058	2.58	
	47.4	(110)	0.019	8.46	
ZIS-Ace	27.5	(102)	0.028	5.29	4.8
	28.9	(104)	0.051	2.96	
	47.5	(110)	0.025	6.25	
ZIS-AceSO_4	27.3	(102)	0.028	5.38	5.7
	28.8	(104)	0.050	2.99	
	47.3	(110)	0.018	8.80	

Table S3 Compare the production values of CO_2RR with others Zinc Indium Sulfide materials.

Semiconductor (precursors)	Method	Morphology	Sacrificial reagent	Light source	CO_2RR	Refs
$ZnIn_2S_4$ ($ZnCl_2$, $InCl_3$, thioacetamide)	Solvothermal (ethylene glycol)	nanosheets		300W Xe lamp	CH_4: 0.232 μmol/g	[1]
$ZnIn_2S_4$ ($ZnCl_2$, $InCl_3$, thioacetamide)	Solvothermal (ethanol)	microspheres	$NaHCO_3$	300W Xe lamp (420nm cut-off)	CH_3OH: 0.3 μmol/g/h	[2]
$ZnIn_2S_4$ ($Zn(NO_3)_2$, $In(NO_3)_3$, thiourea)	Hydrothermal	microspheres<br>cubic<br>hexagonal	methanol	250W Hg lamp (365nm)	$HCOCH_3$: ~200 μmol/g<br>$HCOCH_3$: 629 μmol/g<br>$HCOCH_3$: 762 μmol/g	[3]
$ZnIn_2S_4$ ($Zn(NO_3)_2$, $In(NO_3)_3$, L-cysteine)	Hydrothermal	sheet-like		300W Xe lamp AM1.5G	CO, $V_{zn\text{-}poor}$: 100 μmol/g<br>CO, $V_{zn\text{-}rich}$: 332 μmol/g	[4]
$ZnIn_2S_4$ ($ZnCl_2$, $InCl_3$, thioacetamide)	Solvothermal (50% ethanol)	particles<br>nanosheets	H_2O/acetonitrile $Co(bpy)_3^{2+}$ triethanolamine	300W Xe lamp (400nm cut-off)	$CO_{particles}$: 875μmol/g/h<br>$CO_{nanosheet}$: 1275μmol/g/h	[5]
$ZnIn_2S_4$ ($Zn(Ace)_2$, $In_2(SO_4)_3$, sodium citrate, thioacetamide)	Hydrothermal	nanocrystal	triethanolamine	500W Xe lamp AM1.5G	CO: 1316 μmol/g<br>CH4: 12 μmol/g	**This Work**

Table S4 Compare the production values of CO_2RR with others semiconductor materials.

Semiconductor	Medium	Sacrificial reagent	Light source	CO_2RR	Refs
$CsPbBr_3$ QDs	ethyl acetate/water (15mL/50μL)		300W Xe lamp AM1.5G	CO: 34.1 μmol/g CH_4: 12.2 μmol/g	[6]
$Cs_2AgBiBr_6$ double perovskite	ethyl acetate		100W Xe lamp AM1.5G	CO: 14.1 μmol/g CH_4: 9.6 μmol/g	[7]
$Cs_3Bi_2I_9$ triple perovskite	water vapor		32W UV lamp 305 nm	CO: 77.6 μmol/g CH_4: 14.9 μmol/g	[8]
10 wt% MMT/TiO_2	H_2		200W Hg lamp	CO: 104 μmol/g CH_4: 2.1 μmol/g	[9]
0.03%Bi/$In_2O_{3x}(OH)_y$	H_2		1000 W Hortilux Blue metal halide bulb	CO: 15.84 μmol/g	[10]
Nb_3O_8 nanosheets doped 10% Cu	0.5 M $KHCO_3$ solution (PH = 12)		Hg-Xe lamp (240–300 nm)	CO: 0.07 μmol/h	[11]
Cu_2S nanorod doped Pt	1 M Na_2CO_3		450W Xe lamp	CO: 3.02 μmol/g/h CH_4: 0.13 μmol/g/h	[12]
$ZnIn_2S_4$ ($Zn(Ace)_2$, $In_2(SO_4)_3$, sodium citrate, thioacetamide)	water vapor	triethanolamine	500W Xe lamp AM1.5G	CO: 1316 μmol/g CH_4: 12 μmol/g	**This Work**

Reference

1. Yang, G.; Chen, D.; Ding, H.; Feng, J.; Zhang, J.Z.; Zhu, Y.; Hamid, S.; Bahnemann, D.W. Well-designed 3D $ZnIn_2S_4$ nanosheets/TiO_2 nanobelts as direct Z-scheme photocatalysts for CO_2 photoreduction into renewable hydrocarbon fuel with high efficiency. *Applied Catalysis B: Environmental* **2017**, *219*, 611-618.
2. Mohamed, R.M.; Shawky, A.; Aljandali, M.S. Palladium/zinc indium sulfide microspheres: Enhanced photocatalysts prepare methanol under visible light conditions. *J Taiwan Inst Chem E* **2016**, *65*, 498-504.
3. Chen, J.S.; Xin, F.; Yin, X.H.; Xiang, T.Y.; Wang, Y.W. Synthesis of hexagonal and cubic $ZnIn_2S_4$ nanosheets for the photocatalytic reduction of CO_2 with methanol. *Rsc Advances* **2015**, *5*, 3833-3839.
4. Jiao, X.; Chen, Z.; Li, X.; Sun, Y.; Gao, S.; Yan, W.; Wang, C.; Zhang, Q.; Lin, Y.; Luo, Y.; Xie, Y. Defect-Mediated Electron–Hole Separation in One-Unit-Cell $ZnIn_2S_4$ Layers for Boosted Solar-Driven CO_2 Reduction. *J Am Chem Soc* **2017**, *139*, 7586-7594.
5. Wang, S.; Guan, B.Y.; Lou, X.W.D. Construction of $ZnIn_2S_4$–In_2O_3 Hierarchical Tubular Heterostructures for Efficient CO_2 Photoreduction. *J Am Chem Soc* **2018**, *140*, 5037-5040.
6. Hou, J.; Cao, S.; Wu, Y.; Gao, Z.; Liang, F.; Sun, Y.; Lin, Z.; Sun, L. Inorganic Colloidal Perovskite Quantum Dots for Robust Solar CO_2 Reduction. *Chemistry* **2017**, *23*, 9481-9485.
7. Zhou, L.; Xu, Y.F.; Chen, B.X.; Kuang, D.B.; Su, C.Y. Synthesis and Photocatalytic Application of Stable Lead-Free $Cs_2AgBiBr_6$ Perovskite Nanocrystals. *Small* **2018**, *14*, e1703762.
8. Bhosale, S.S.; Kharade, A.K.; Jokar, E.; Fathi, A.; Chang, S.M.; Diau, E.W. Mechanism of Photocatalytic CO_2 Reduction by Bismuth-Based Perovskite Nanocrystals at the Gas–Solid Interface. *J Am Chem Soc* **2019**, *141*, 20434-20442.
9. Tahir M., T.B., Amin N. Photocatalytic Reverse Water Gas Shift CO_2 Reduction to CO over Montmorillonite Supported TiO_2 Nanocomposite. *CHEMICAL ENGINEERING TRANSACTIONS* **2017**, *56*, 319-324.
10. Dong, Y.; Ghuman, K.K.; Popescu, R.; Duchesne, P.N.; Zhou, W.; Loh, J.Y.Y.; Jelle, A.A.; Jia, J.; Wang, D.; Mu, X.; et al. Tailoring Surface Frustrated Lewis Pairs of $In_2O_{3-x}(OH)_y$ for Gas-Phase Heterogeneous Photocatalytic Reduction of CO_2 by Isomorphous Substitution of In^{3+} with Bi^{3+}. *Adv Sci (Weinh)* **2018**, *5*, 1700732.

11. Yin, G.; Nishikawa, M.; Nosaka, Y.; Srinivasan, N.; Atarashi, D.; Sakai, E.; Miyauchi, M. Photocatalytic carbon dioxide reduction by copper oxide nanocluster-grafted niobate nanosheets. *ACS Nano* **2015**, *9*, 2111-2119.
12. Manzi, A.; Simon, T.; Sonnleitner, C.; Doblinger, M.; Wyrwich, R.; Stern, O.; Stolarczyk, J.K.; Feldmann, J. Light-induced cation exchange for copper sulfide based CO_2 reduction. *J Am Chem Soc* **2015**, *137*, 14007-14010.
